# Editorial: Exploring Bacterial Colonies in Solid Foods or Model Foods Using Non-Destructive Techniques

**DOI:** 10.3389/fmicb.2015.01440

**Published:** 2015-12-22

**Authors:** Sophie Jeanson, Anne Thierry

**Affiliations:** ^1^Institut National de la Recherche Agronomique, UMR1253, Science and Technology of Milk and EggsRennes, France; ^2^AGROCAMPUS OUEST, UMR1253, Science and Technology of Milk and EggsRennes, France

**Keywords:** bacterial colonies, growth, physiology, solid foods, modeling, non-destructive techniques

Bacteria are present in all foods, whether they are indigenous or inoculated. They can be beneficial to the quality of foods, responsible for food spoilage, or even pathogens. In solid food products, bacteria are immobilized. They thus grow as colonies entrapped within the food products or on the food surfaces. In both cases, bacteria interact with the solid matrix, sometimes facing difficulties to access the nutrients as nutrients have to diffuse from the matrix to the bacterial colonies. Bacterial development can thus be impaired in solid matrices in comparison to planktonic growth. To control the growth of bacteria in solid foods is then of major importance. In the case of pathogens, it is crucial for safety issues to predict how the bacteria present as initial contaminants will develop. In the case of inoculated bacteria, such as lactic acid bacteria, it is also crucial to control their development because they are responsible for the final quality of the food products. However, studies on the growth of bacteria have been essentially focused on growth in liquid media. Resolute techniques, which include both microcopy approaches and quantitative techniques, have now been developed to observe colonies at the microscopic scale. They allow studying the variation of growth in different contexts either in model growth culture media or in model foods.

This Research Topic starts with four review papers. A first comprehensive review about bacterial colonies redraws the history of the studies on colonies, synthetizes the conditions of growth in which growth in colonies differs from planktonic growth, and finally presents concepts of the interaction of bacterial colonies with the food matrix in which they grow, with cheese as an example (Jeanson et al.). The second review demonstrates the importance of modeling the growth of bacterial colonies in solids foods despite a lack of knowledge. The different types of models and their potential consequences on decisions are presented and discussed (Skandamis and Jeanson). The third review shows how non-invasive techniques can be used to observe bacterial growth and also quantify the bacterial metabolism in colonies at the microscopic scale (Lobete et al.). The last review paper shows how microscopy techniques are particularly valuable to increase knowledge about bacterial colonies in model foods and foods. Fluorescent microscopy allows targeting metabolites and understand the physiology of bacteria within colonies (Hickey et al.).

The five following papers present original results in the field. Using an automated microscope coupled with fluorescence dyes, it was possible to demonstrate that the exposure to an oxidizing disinfectant led to different morphologies of cells depending on the strains and that dead cells were randomly distributed within the micro-colonies (Zhao et al.). The following study demonstrates the accuracy of a phenotyping technology based on laser-induced speckle scatter patterns in *Bacillus* colonies. The authors showed that the distribution of speckle size is modified during the growth of colonies (Kim et al.).

The three following studies investigate bacterial colonies of lactic acid bacteria in their food environment in model cheeses or investigate the pH in their surrounding micro-environment in Cheddar cheeses. Physiology and growth of *Streptococcus thermophilus* colonies was compared in liquid milk and renneted milk using microcalorimetry. The results showed that above a threshold size of colonies (low inoculation levels), growth rates and metabolism in renneted milk differed from the one observed in liquid milk (Stulova et al.). The use of fluorescence lifetime imaging was shown to be relevant to investigate the pH micro-heterogeneity in Cheddar cheeses (Burdikova et al.). These results suggest that the surroundings of bacterial colonies within cheeses could differ from homogeneous growth conditions in liquid medium. The last study questioned the access of bacteria in colonies to their nutrients, through the assessment of diffusion of solutes inside colonies. The diffusion of high molecular weight molecules was assessed inside *Lactococcus lactis* colonies grown in a model cheese. The results led to the conclusion that diffusion of molecules inside bacterial colonies depends on the physicochemical properties of the molecules (Floury et al.).

In conclusion, since bacteria mainly grow in colonies in foods, increase knowledge on growth and physiology of bacteria growing as colonies is now a crucial issue. This is achieved by the recent development of non-invasive techniques that allows investigating at the microscopic scale with time lapse and quantitative analyses. Knowledge about the growth and the adaptive response of bacteria to the food environment will continue to grow by addressing the remaining questions about interactions between bacterial colonies and their food environment.

## Author contributions

SJ: wrote the editorial. AT: revised and improved the editorial.

### Conflict of interest statement

The authors declare that the research was conducted in the absence of any commercial or financial relationships that could be construed as a potential conflict of interest.

